# Isomerized Aβ in the brains can distinguish the status of amyloidosis in the Alzheimer’s Disease continuum

**DOI:** 10.1002/alz.090569

**Published:** 2025-01-03

**Authors:** Soumya Mukherjee, Celine Dubois, Keyla Perez, Ian Birchall, Fairlie Hinton, Catriona McLean, Colin L Masters, Blaine Russell Roberts

**Affiliations:** ^1^ Washington University School of Medicine, St. Louis, MO USA; ^2^ The Florey Institute of Neuroscience and Mental Health, PARKVILLE, VIC Australia; ^3^ The University of Melbourne, Parkville, VIC Australia; ^4^ The Florey Institute of Neuroscience and Mental Health, Parkville Australia; ^5^ The Florey Institute of Neuroscience and Mental Health, Melbourne, VIC Australia; ^6^ Victorian Brain Bank Network (VBBN), Melbourne, VIC Australia; ^7^ Florey Institute of Neuroscience and Mental Health, University of Melbourne, Parkville, VIC Australia; ^8^ Emory University School of Medicine, Atlanta, GA USA

## Abstract

**Background:**

The extracellular amyloid plaques, one of the pathological hallmarks of Alzheimers Disease (AD), are frequently also observed in the cortex of cognitively unimpaired subjects or as co‐pathology in other neurodegenerative diseases. Progressive deposition of fibrillar amyloid‐β (Aβ) as amyloid plaques for two decades prior disease onset leads to extensive isomerization of Aβ N‐terminus. Quantifying the extent of isomerized Aβ can be provide insight into the different stages of amyloidosis in the brain.

**Methods:**

We measured Aβ isoforms using mass spectrometry. We analysed postmortem brains (frontal cortex) from patient with AD (n = 59), Parkinson disease (n = 57) and age matched control brains (61). We also measured Asp residue isomerization at Aβ N‐terminus.

**Results:**

We observed a significant increase in isomerization of Aβ N‐terminus in AD compared to age‐matched control brains in the soluble and insoluble pool.^1‐3^ While AD brains contain significant amount of Asp‐1 and Asp‐7 isomerized Aβ_1‐15,_ cognitively unimpaired patients with amyloid deposits (CU‐AP) also have minor isomerized species. We further demonstrate that ratio of isomerized N‐terminus Aβ (Aβ_1‐15_) species in the brain soluble pool differentiates amyloid deposits in AD brain compared to CU‐AP individuals and patients with co‐morbidities.

**Conclusions:**

Isomerized N‐terminus Aβ (Aβ_1‐15_) is a common feature of parenchymal amyloid deposits in the brain. Our results suggest Aβ deposits associated with early AD neuropathologic changes are distinct to the mature plaques found in AD, a consequence of prolonged deposition in the later. Our results have implications for early therapeutic targeting of these modified species as well potential for better biofluids biomarker development to track drug efficacy.

**References**

1. Mukherjee S, Perez KA, Lago LC, et al. Quantification of N‐terminal amyloid‐β isoforms reveals isomers are the most abundant form of the amyloid‐β peptide in sporadic Alzheimer’s disease. *Brain Commun*. 2021;3(2). https://doi.org/10.1093/braincomms/fcab028

2. Mukherjee S, Perez KA, Dubois C, et al. Citrullination of Amyloid‐β Peptides in Alzheimer’s Disease. *ACS Chem Neurosci*. 2021;12(19):3719‐3732. https://doi.org/10.1021/acschemneuro.1c00474

3. Mukherjee S, Fjeldsted JC, Masters CL, Roberts BR. Enhanced ion mobility resolution of Abeta isomers from human brain using high‐resolution demultiplexing software. *Anal Bioanal Chem*. Published online April 15, 2022. https://doi.org/10.1007/s00216‐022‐04055‐x

